# Status of imported malaria on Réunion Island in 2016

**DOI:** 10.1186/s12936-018-2345-y

**Published:** 2018-05-24

**Authors:** Frédéric Pagès, Sandrine Houze, Brian Kurtkowiak, Elsa Balleydier, François Chieze, Laurent Filleul

**Affiliations:** 1 Santé Publique France, Cire Océan Indien, 2, bis avenue Georges Brassens-CS 61002, 97713 Saint-Denis Cedex 9, Saint-Denis, Réunion France; 2 CNR Paludisme-Laboratoire de Parasitologie, APHP, Hôpital Bichat, Paris, France; 3 UMR 216, Merit, Faculté de Pharmacie, Université Paris Descartes, Paris, France; 4 Vector Control Department, Agence régionale de santé Océan Indien, Paris, Saint-Denis, Réunion France; 5 Health Monitoring Department, Agence régionale de santé Océan Indien, Paris, Saint-Denis, Réunion France

**Keywords:** Imported malaria, Reunion Island, Indian Ocean, Chimio-susceptibility, Travellers

## Abstract

**Background:**

Autochthonous malaria has been eliminated from Réunion in 1979. To prevent secondary transmission and re-emergence of autochthonous malaria, permanent epidemiologic and entomological surveillance and vector control measures are conducted around imported malaria cases. Results of local malaria surveillance (clinical data and results of epidemiological and entomological investigations around cases) were collected for 2013–2016 and were analysed according to historical data and to the exchanges with malaria-affected areas (estimated by airport data).

**Results:**

Form 2013 to 2016, 95 imported malaria cases have been detected in Reunion Island: 42% of cases occurred in the area of repartition of *Anopheles arabiensis,* but Anopheles mosquitoes were present only around seven cases including one gametocyte carrier. No autochthonous or introduced case has occurred during this period. The lack of chemoprophylaxis or poor adherence was found in the majority (96%) of malaria cases between 2013 and 2016, regardless of trip type. Affinity tourism in Madagascar and Comoros was the cause of 65% of imported malaria cases.

**Discussion:**

The incidence of imported malaria and the incidence rate per 100,000 travellers has continuously decreased since 2001. Now with the drastic decrease of malaria transmission in the Comoros archipelago, most of imported malaria cases in Reunion Island have been contaminated in Madagascar. Immigrants regularly resident in Reunion Island, which travel to malaria endemic countries (mainly Madagascar) to visit their friends and relatives (VFRs) represent a high-risk group of contracting malaria. VFRs, low adherence to pre-travel recommendations, in particular, the compliance on the use of chemoprophylaxis are the main drivers of imported malaria in Reunion Island. Furthermore as previously described, some general practitioners in Reunion Island are always not sufficiently aware of the official recommendations for prescriptions of prophylactic treatments.

**Conclusion:**

Social mobilization targeted on the Malagasy community in Reunion Island could help to decrease the burden of imported malaria in Reunion Island. Because of the low number of gametocyte carriers and the absence of an *Anopheles* mosquito population when most malaria cases were imported those last 4 years, the risk of the appearance of introduced malaria cases and indigenous malaria cases appears low in Reunion Island.

## Background

Réunion Island is a French overseas department in the south-western Indian Ocean. The island is located between Madagascar and Mauritius. As of 2013, it had a population of 840,000. There are regular exchanges between Réunion and the surrounding islands, including the Union of the Comoros, Madagascar, Mauritius, Mayotte, and Seychelles. Malaria arrived in Réunion in 1869 and quickly became endemic until DDT started being spraying inside homes in 1948. The eradication of malaria can be achieved and maintained through larval control programmes and increased epidemiological surveillance [1]. The last malaria cases of indigenous malaria in Réunion were reported in 1967. The World Health Organization (WHO) declared that malaria had been eliminated from the island in 1979 [2].

Due to the presence of an effective vector (*Anopheles arabiensis*), the island is considered potentially vulnerable to the reintroduction of malaria, or at least introduced malaria cases (i.e. an infection acquired in Réunion as a direct result of an imported case) or even an indigenous case (i.e. an infection acquired in Réunion after the formation of a local transmission chain beginning with an imported case) [3]. Since 2000, three introduced malaria cases of malaria have been reported to the health authorities. The last case occurred in 2006 [2]. As a result, a special epidemiological surveillance programme has been created in Réunion specifically for malaria in addition to the national surveillance programme for imported malaria cases [4, 5]. The main goals of this surveillance programme are to prevent introduced cases of malaria and the reoccurrence of indigenous transmission by intervening in each case of imported malaria and detecting every potentially introduced or indigenous case as soon as possible. The secondary goals of the programme are to describe the clinical characteristics and the prevention practices surrounding malaria infections occurring in people travelling to Réunion. Each case of malaria must be declared to the Regional Health Agency (Agence Régionale de Santé—ARS), triggering an epidemiological and entomological investigation by the Vector Control Service (Service de lutte antivectorielle—LAV) that could be followed by anti-mosquito operations. Since malaria was eradicated in Réunion, anopheline (mostly *An. arabiensis*) distribution has shrunk. *Anopheles* mosquitoes are present only in certain regions on the island [6]. In addition, their density is not conducive to transmission except in a small area during certain periods of the year [6]. Once malaria transmission had been stopped in Réunion, the majority of imported malaria cases came from Réunion residents returning from holidays, either for tourism purposes or to visit family and friends and relatives (VFRs), in the surrounding regions, i.e. Comoros, Madagascar, and Mayotte [7]. Several past studies have shown that a certain number of these malaria cases could be due to a low rate of medical consultation before departure, a low rate of compliance with prevention measures (chemoprophylaxis and personal protection from disease vectors), and inappropriate advice and medication issued by the medical community in Réunion [7, 8].

In the past few years, the malaria situation in the surrounding region has changed due to the successes gained through the anti-malaria campaigns in the Comoros Archipelago (Union of Comoros and Mayotte) and, to a lesser extent, Madagascar [9–11]. At the same time, due to the increase in tourism and international exchange, the possibility of an imported case from other world regions has increased. Below, we present the epidemiological surveillance report for imported malaria in Réunion from 2013 to 2016. The main goal of this report is to describe the clinical and epidemiological characteristics of these cases, patients’ prevention practices (warnings before travel, chemoprophylaxis, and vector protection measures), and indirectly whether or not the chemoprophylaxis prescriptions provided are in line with recommendations. The secondary goal is to assess the risk of the reintroduction of malaria over the past 4 years by studying data from entomological investigations regarding malaria cases and time of import as well as the presence of gametocytes in blood smears.

## Methods

In Réunion Island, all public or private laboratories and all physicians have to report immediately all confirmed malaria cases to the regional health agency that triggers an epidemiological investigation to determine the place and circumstances of contamination and an entomological investigation to assess the risk of secondary cases due to local population of *An. arabiensis*. The report used epidemiological surveillance data for malaria infections in Réunion. Previous raw data (number of malaria cases and areas of contamination) were collected. Airport data from the island’s only international airport located in St. Denis were also collected to estimate the number of passengers arriving from malaria-affected regions every year. An overall incidence rate per 100,000 travellers from malaria-affected regions was calculated for 2000–2016. Specific incidence rates were also calculated for each travel destination between 2013 and 2016 as well as the season (winter and summer in the Southern Hemisphere) in each travel destination between 2013 and 2016.

To confirm that the number of reported malaria cases was accurate, the four hospital laboratories were asked to provide an anonymized list of the malaria diagnoses from the previous year at the start of each year. The data were cross-referenced with the reported cases of malaria according to age, sex, month of diagnosis, and town of residence. When a case was not reported, demographic, clinical, biological, and epidemiological information, as well as information regarding prevention practices, if possible, was gathered from the medical professional who treated the patient. In France, rapid diagnosis tests are considered as medical device and not available for self-diagnosis.

Data from ARS investigations between 2013 and 2016 were used. Each case led to an in-home investigation and the creation of a report that included demographic data (age, sex, place of residence, nationality, and profession), clinical data (uncomplicated or severe case, hospitalization or out-patient care, and treatment prescribed), biological data (diagnostic methods, *Plasmodium* species, parasitaemia, presence or absence of sexual forms), epidemiological data (place of travel, type of travel, dates, and duration of travel), prevention practices data (medical consultation before travelling, prescription of chemoprophylaxis, compliance with chemoprophylaxis and anti-vectorial personal prevention, and causes of the lack or poor quality of adherence), and entomological data. As part of the surveillance programme, each reported case is always followed by a search for *Anopheles* larvae. Entomological data is collected when the *Anopheles* mosquitoes are active at the patient’s place of residence and in the places where he or she has spent time since returning to Réunion, including the patient’s workplace if necessary. Along with entomological investigations surrounding malaria cases, Réunion has a longitudinal entomological surveillance programme in areas where *Anopheles* mosquitoes are present. Adulticides and larvicides are applied if the entomological investigation finds *Anopheles* mosquito larvae or if longitudinal monitoring efforts detected the presence of *Anopheles* mosquitoes in the previous weeks in areas where these types of mosquitoes are present.

As part of the national surveillance programme for imported cases of malaria, it is recommended to monitor the susceptibility of *Plasmodium falciparum* to anti-malarial drugs used to treat or prevent the disease. Strains must be sent to the National Malaria Reference Centre (CNR Paludisme). In 2013, this procedure was again initiated in Réunion Island. Each laboratory was asked to send an EDTA tube of blood stored at 4 °C to the National Malaria Reference Centre in Paris. On blood samples, malaria diagnosis was confirmed; species and parasitaemia were determined by microscopy. In case of no *Plasmodium falciparum* parasites, qPCR was performed to confirm the species (FTD Malaria differentiation, Fast Track Diagnostics). The goal was to carry out in vitro testing to determine the susceptibility of *P. falciparum* strains to the anti-malarials used for chemoprophylaxis and treatment, look for molecular markers indicating anti-malarial resistance [12]. The susceptibility of *P. falciparum* was evaluated using the ex vivo isotopic microtest as previously described [13]. Susceptibility or resistance were assessed for mono desethylamodiaquine, lumefantrine, piperaquine, dihydroartemisinin, chloroquine, quinine, mefloquine and doxycycline. Chloroquine, mono desethylamodiaquine, lumefantrine, piperaquine, pyronaridine, quinine, mefloquine, dihydroartemisinin, artesunate and doxycycline were distributed into 96-well plates as previously described. The 50% inhibitory concentration (IC50), defined as the drug concentration corresponding to 50% of the uptake of 3H-hypoxanthine measured in the drug-free control wells, was determined by Ice Estimator software [14].

For the analysis of molecular markers, total genomic DNA was extracted from blood sample using a QIAamp DNA Blood Mini Kit according to the manufacturer’s recommendations (Qiagen, Germany). In 2013, 2015 and 2016, crtK76T by the PCR–RFLP as previously described and cytb 268 SNPs were determined. In 2013, isolates were genotyped for *dhfr* SNPs at codons 51, 59, and 108, as previously described. In addition, in 2015 and 2016, mdr1 SNPs 86, 184, and 1246 and PfK13 genotyping were determined as described [15–19]. In case of cluster cases, five *P. falciparum* microsatellite loci (TAA 81, TAA 87, TAA 60, PKPK 1, and ARA 2) located on different chromosomes were genotyped using the method described by Musset et al. [4].

### Statistics

The anonymized forms were entered in Epidata, and the data were analysed with Epiinfo version 7. Percentages were compared using the Khi^2^ test, and quantitative data were compared with the Student or Wilcoxon tests.

## Results

### Evolution of imported malaria in Réunion Island between 2013 and 2016

In 2013, 40 cases of imported malaria were reported in Réunion. Thirty-five malaria cases were diagnosed on the island, and five severe malaria cases diagnosed in countries in the surrounding region or on ships circulating in the area required medical evacuation for treatment, including one sailor who had come ashore in Cameroon 1 month before symptom onset and four patients evacuated from Madagascar, three of which were French nationals residing in Madagascar. In 2014, 2015, and 2016, 18, 27, and 12 cases of imported malaria, respectively, were reported in Réunion, including three evacuated patients from Madagascar. An examination of laboratory hospital data uncovered five unreported malaria cases (including one severe infection) in 2013, bringing the total number of malaria cases to 45, and one unreported case in 2014 for a total of nineteen malaria cases. In 2015 and 2016, no unreported malaria cases were found. The exhaustivity of malaria surveillance was above 88.8% for 2013 and 94.7% in 2014. It reached 100% in 2015 and 2016, assuming every case treated by doctors outside of a hospital setting was reported. Between 2013 and 2016, 103 cases of malaria were treated in Réunion Island. During this 3-year period, the exhaustivity of malaria surveillance was 94%. Excluding medically evacuated malaria cases, over a third of malaria infections (33) was diagnosed and treated on an outpatient basis.

**Fig. 1:**
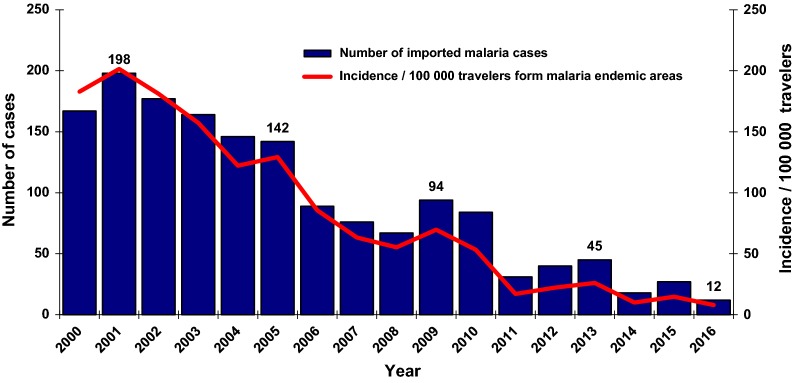
Annual distribution of imported malaria cases and the evolution of the incidence rate per 100,000 travellers arriving from malaria-affected regions, Réunion, 2000–2016 (n = 1565)

From 2001 to 2016, the number of imported malaria cases was reduced by a factor of 16 in Réunion (from 198 in 2001 to 12 malaria cases in 2016) even though the number of travellers arriving directly from regions affected by malaria (Comoros, Madagascar, Africa, and India) nearly doubled (increasing from 98,271 in 2001 to 150,684 in 2016). The rate of incidence among travellers from malaria-risk regions has been divided by 25, dropping from over 200 malaria cases per 100,000 travellers in 2001 to around eight malaria cases per 100,000 travellers in 2016 (Fig. 1).

The yearly incidence rate for imported malaria infections per 100,000 travellers between 2013 and 2016 was calculated by comparing the number of imported malaria cases (excluding medically-evacuated patients) to the number of travellers from malaria-risk regions. Between 2013 and 2016, the incidence rates were 23.1, 10.6, 14.1, and 6.6 per 100,000 travellers, respectively. The incidence rate for each country of destination was calculated for the Union of Comoros, Mayotte, India, and Madagascar (Table 1). Between 2013 and 2016, the yearly malaria incidence rate among travellers arriving from the Union of Comoros dropped significantly, whereas the incidence rate for Madagascar remained stable. For infections contracted during a stay on the African continent, because of the lack of direct flights between African countries and Réunion (except South Africa), information regarding these travellers is limited outside of those arriving directly from South Africa.

**Table 1:** Imported malaria incidence rate per 100,000 travellers according to country of origin, Réunion, 2013–2016

	Annual incidence/100,000 travellers (number of malaria cases)				
	India	Union of Comoros	Mayotte	Madagascar	All malaria-affected areas
2013	106.6 (1)	472.9 (22)	0 (0)	25.7 (17)	23.1 (40^a^)
2014	54.8 (1)	36.7 (2)	1.2 (1)	14.7 (10)	10.6 (19)
2015	0 (0)	0 (0)	0 (0)	29.1 (19)	14.1 (26^a^)
2016	0 (0)	0 (0)	0 (0)	9.1 (10)	6.6 (10^a^)

^a^Excluding medically evacuated patients

**Fig. 2:**
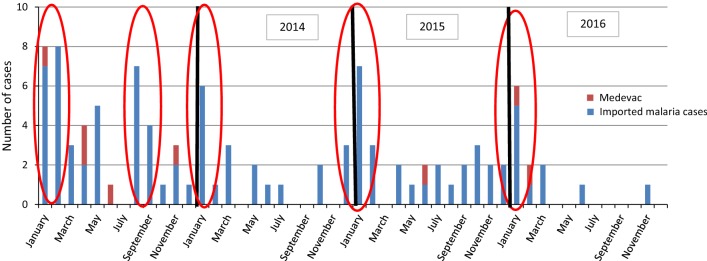
Monthly distribution of malaria cases treated on Réunion Island, 2013–2016 (n = 103)

The monthly distribution of malaria cases between 2013 and 2016 is presented in Fig. 2. Cases of malaria occur after the school holidays. Most malaria cases occur after the two largest holidays, namely Christmas and the end of the year, but malaria cases also occur during the May holiday. The incidence rate for imported malaria infections is higher following the holidays, especially during the austral summer holiday (Table 2).

**Table 2:** Imported incidence rate per 100,000 travellers according to country of origin and period of year, Réunion, 2013–2016

	Union of Comoros	Madagascar	All malaria-affected areas
2013			
Annual IR	472.9	25.7	23.1
Summer IR	917.4	40.5	51.2
Winter IR	607.3	8.3	33
2014			
Annual IR	36.7	14.7	10.6
Summer IR	277.4	40.1	22.6
Winter IR	0	0	0
2015			
Annual IR	0	29.1	14.1
Summer IR	0	70.2	31.5
Winter IR	0	16.6	7.9
2016			
Annual IR	0	6.6	9.1
Summer IR^a^	0	28.9	11.8
Winter IR	0	0	0

*IR* incidence rate per 100,000 travelers

^a^Including imported malaria cases from January to February 2017

### Circumstances and country of occurrence

**Fig. 3:**
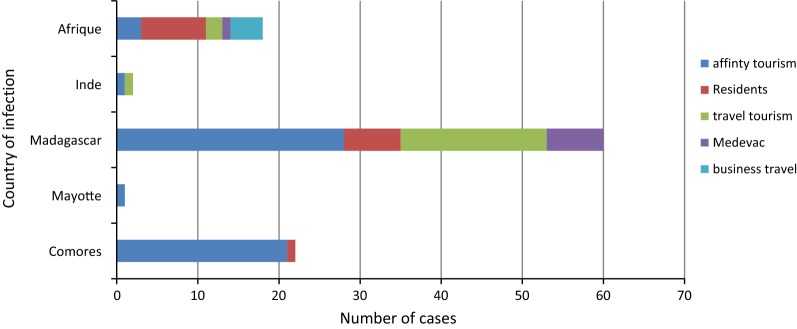
Distribution by country of infection and trip type in endemic regions of imported malaria cases occurring in Réunion (n = 95) and malaria cases medically evacuated from the surrounding region (n = 8), Réunion, 2013–2016

Concerning the 95 malaria cases that occurred in Réunion, 21 involved travel tourism: eighteen *P. falciparum* infections after returning from Madagascar, two *P. falciparum* infection after returning from West Africa, and one *P. vivax* and *P. falciparum* infection after returning from India); 54 involved affinity tourism (travel to VFRs): 21 *P. falciparum* infections after returning from the Comoros archipelago, including one patient who returned from Mayotte, 25 people returning from Madagascar after staying with people from the surrounding region of French nationality or otherwise, one *P. falciparum* infection after returning from India, two *P. falciparum* infection after returning from central Africa, one *P. falciparum* infection after returning from West Africa, two *P. vivax* (Madagascar and Comoros) infections, one *Plasmodium malariae* and *P. falciparum* (Madagascar) infection, one *P. malariae* and *P. vivax* (Madagascar) infection; eight infections involving Réunion residents who spent over 6 months per year in an island in the surrounding region (seven in Madagascar, one in Union of Comoros); eight *P. falciparum* infections in people living in endemic areas (four in Central Africa, one in West Africa, one in Guyana, and one in Madagascar), and four *P. falciparum* infections contracted during a business trip (two in Central Africa and one in West Africa). The distribution of malaria cases according to the countries in which the infection was contracted and trip type between 2013 and 2016 is presented in Fig. 3.

### Case characteristics

**Fig. 4:**
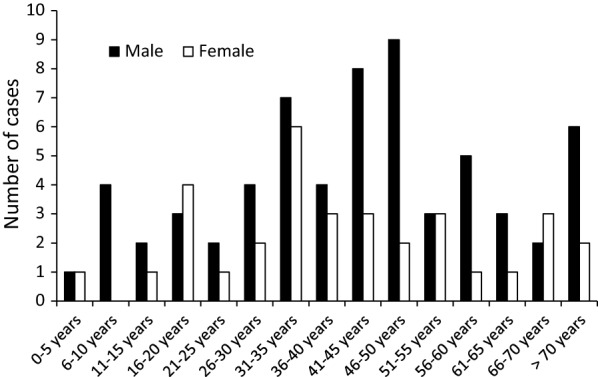
Distribution by sex and age group of imported malaria cases occurring in Réunion and the six malaria cases medically evacuated from the surrounding region, Réunion, 2013–2016 (n = 103)

Thirty-four women (four severe infections) and 69 men (11 severe infections including two children aged three and eight) were infected. The average age of the women was 40.5 years (range of 4 to 77 years old) compared to 43 for the men (range of 3 to 79 years old). The distribution of malaria cases by sex and age group is presented in Fig. 4.

### Parasitological diagnosis

The majority of diagnosis (95/103) was done by observing a blood smear under a microscope. Four were confirmed using only the malaria rapid diagnostic test, which looks for plasmodial antigens, and three malaria cases were diagnosed using only molecular blood tests (qPCR). The majority of infections was caused by *P. falciparum* (91 malaria cases including seven medically evacuated patients), followed by *P. vivax* (three infections including one medically evacuated patient), and *Plasmodium ovale* (three patients). Two infections were caused by *P. malariae,* one case was caused by a co-infection of *P. falciparum* and *P. malariae*, one case was caused by a co-infection of *P. malariae* and *P. vivax,* and in one case, the *Plasmodium* species was not identified. Among the diagnoses made by microscopy, gametocytes were found in 6 out of 95 patients: four *P. falciparum* infections (including one medically-evacuated patient), one co-infection by *P. malariae* and *P. vivax,* and one co-infection by *P. vivax* and *P. falciparum*.

### Clinical forms

In total, 88 uncomplicated infections (including three medically evacuated patients) and 15 severe infections (including five medically evacuated patients) were treated on the island between 2013 and 2016. Excluding the five patients who were medically evacuated to Réunion for treatment, including one patient from Madagascar who died in 2013, ten severe forms of *P. falciparum* infection occurred in Réunion. Five malaria cases were due to travel tourism, including one patient who died in 2015, two malaria cases to affinity tourism, one case to a business trip and two malaria cases occurred in Madagascar residents.

**Fig. 5:**
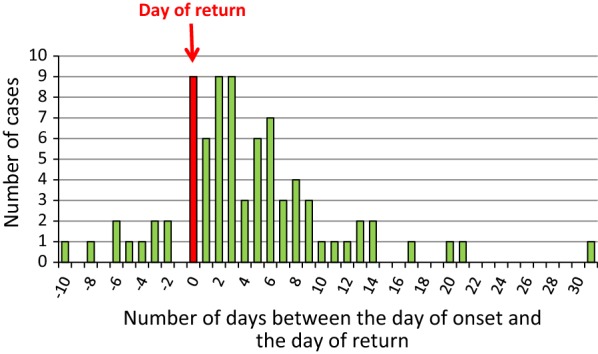
Delay between the first signs of infection and the date of arrival in Réunion for the *P. falciparum* malaria cases, Réunion, 2013–2016 (n = 80)

For single or co-infections involving *P. falciparum*, the date of first symptom onset was available for eighty malaria cases (excluding medically evacuated patients). Clinical signs occurred while the patient was still in the country of infection for ten patients. For the other patients, the median length of time until the appearance of the first clinical signs compared to the patient’s date of return from the endemic country was 6 days (ranging from 0 to 31 days). The distribution of malaria cases according to the patient’s date of return is provided in Fig. 5. Regarding the two cases of malaria caused by *P. malariae*, the first signs of disease appeared 72 days after for one case. No data was available for the second case. Concerning *P. vivax* infections, the clinical signs first appeared prior to the patient’s return in one case, 7 days after returning for two malaria cases, and 537 days after returning in one case (travelled to Grande Comore in March 2012 and the infection occurred in September 2013). Regarding *P. ovale* infections, clinical signs started the day after the patient returned to Réunion in one case, 93 days after for the second, and 278 days for the last. For species that can cause relapse due to quiescent hepatic parasite stages, the delay between the patient’s return from an endemic region and the appearance of clinical signs can vary widely.

### Susceptibility to anti-malarial medications

Twenty strains (the total for 2013) were sent to the National Malaria Reference Centre by hospital and non-hospital laboratories. Due to transport time, no IC50 determination could be performed. Only molecular genotypes linked with anti-malarial resistance were determined. No mutations in *pfcyt b* was observed and all *P. falciparum* isolates were sensitive to atovaquone, included in the combination of atovaquone and proguanil (atovaquone/proguanil); 16.7% of isolates presented the mutation *Pfcrt* 76T and were considered as resistant to chloroquine (isolates from Comoros representing 37.5% of isolates from Comoros); and 42.9% of isolates presented the triple 51, 59 and 108 *dhfr* gene mutations associated with a decreased sensitivity to proguanil (2 isolates from Madagascar representing 33.3% of isolates from Madagascar and 2 isolates from Comoros representing 66.6% of isolates from Comoros). In 2015 and 2016, no mutation in *Pfk13* gene associated with artemisinin resistance was observed. Among the six isolates received in 2015, only the *Pfmdr* 1 184F mutation was observed in four isolates, *Pfmdr1* 86 and 1246 positions were wild-type.

### Chemoprophylaxis use

Out of the 95 malaria cases that occurred in Réunion, data regarding chemoprophylaxis use was available for 89 patients. Twenty-nine patients were prescribed chemoprophylaxis: ten received chloroquine, seven received atovaquone/proguanil, four received the combination of chloroquine and proguanil (Chloroquine/proguanil), three received mefloquine, two received doxycycline, and four patients did not remember the name of the medication they were prescribed. Out of these patients, ten did not buy the medication and, therefore, did not comply with the prescription, 13 stopped taking their medication early, three reported taking their medication irregularly, and only five reported proper adherence: one patient took chloroquine (*P. falciparum* infection), two patients took atovaquone/proguanil (one *P. malariae* infection and one *P. ovale* infection while patients were on medication), and one patient was on mefloquine (relapse of *P. vivax* occurring over 1 year after the patient’s return). Fifty-six patients were not prescribed an anti-malarial medication. Out of these patients, 24 were not aware that they should have consulted a doctor, 21 chose to not consult a doctor, and eleven were not prescribed a medication after their consultation, including one 2-year-old child whom the physician considered to be too young to take the medication, one patient who did not receive a prescription because he was only staying in an urban environment in India, and another was prescribed atovaquone/proguanil, but as a presumptive treatment to take in the case of fever during the patient’s stay. An absence of chemoprophylaxis or poor adherence was found in the majority of malaria cases (96%) regardless of the reason for travel (visiting friends and family or for other reasons: 93% vs. 88%, i.e. a non-significant difference). Chemoprophylaxis prescriptions (whether or not they were bought and/or taken) were inappropriate in over half the malaria cases (52%) with respect to the recommendations for the countries visited. The anti-malarial quantitative analyses conducted at the National Malaria Reference Centre matched what patients had reported, including patients who did not take their medication or stopped early. Irregular use of chloroquine/proguanil was confirmed through blood analysis of two patients, one of which reported recent and premature cessation and another who reported irregular use.

### Treatment

Out of 103 patients, thirty-three were treated on an outpatient basis and 70 patients were hospitalized, including eight medically-evacuated patients. The average length of hospital stay was longer for severe malaria cases (*p *= 0.0005, Wilcoxon test): 19 days (median of 11 days) compared to 6 days for uncomplicated malaria cases (median of 3 days). Out of the 33 patients treated on an outpatient basis, ten were treated with a combination of atovaquone and proguanil (atovaquone/proguanil), 13 with a combination of artemether and lumefantrine, six by chloroquine (Nivaquine^®^) (including one for a *P. vivax* infection and one for a *P. malariae* infection), one by oral quinine, and two by mefloquine. The treatment was unknown for one patient. Regarding the hospitalized patients, 24 were treated by artemether/lumefantrine, 11 by atovaquone/proguanil, five by quinine, four by Nivaquine^®^ (three for a *P. vivax* infection and one for a *P. malariae* infection), seven by quinine followed by artemether/lumefantrine, eight by injectable artesunate followed by artemether/lumefantrine, one by mefloquine, one by quinine then injectable artesunate then artemether/lumefantrine, and one by artemether/lumefantrine then primaquine. The treatment was unknown for two patients.

### Geographic distribution of malaria cases

**Fig. 6:**
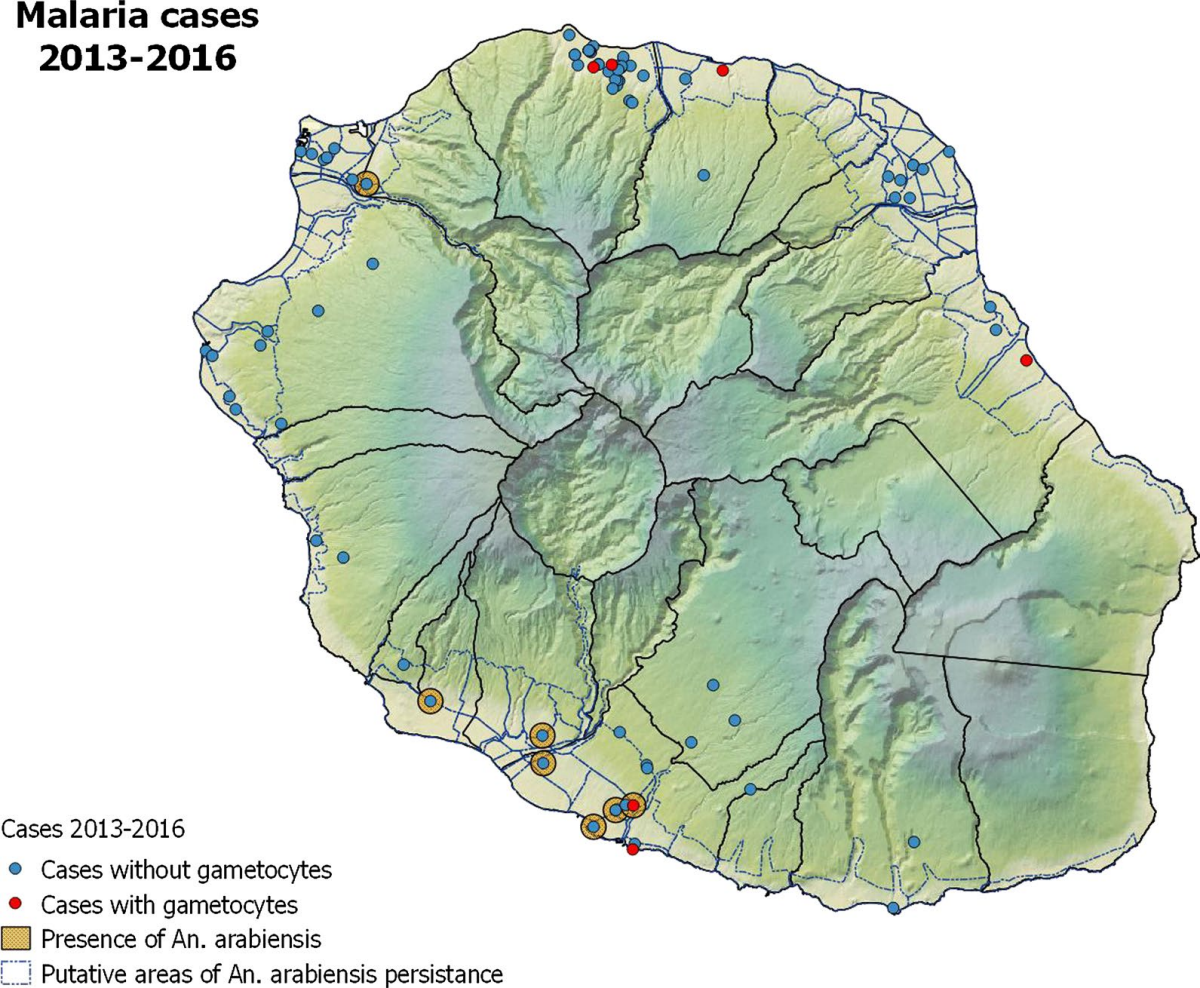
Geographic distribution of malaria cases, presence of gametocytes in blood smear and *Anopheles* mosquitoes upon diagnosis, Réunion, 2013–2016

The geographic distribution of malaria cases between 2013 and 2016 is provided in Fig. 6. Between 2013 and 2016, for the ninety-one malaria cases that were the subject of an entomological investigation, *Anopheles* mosquitoes were found at the time of the malaria infection in only seven malaria cases in which gametocytes were not present in patients during diagnosis: two in 2013, two in 2015 and three in 2016. Gametocytes were found in only seven patients: six concerned imported malaria cases (three *P. falciparum* infections, two *P. vivax* infections, and one *P. malariae* and *P. vivax* co-infection), and one case concerned a patient who was medically-evacuated from a ship. Out of the 95 cases of imported malaria in Réunion, only forty (42%) occurred in areas in which the *Anopheles* mosquito persists. In 4 year, *Anopheles arabiensis* were found only one time around a patient carrier of gametocytes.

### Cluster of malaria cases

Between 2013 and 2016, most of reported malaria cases were sporadic. Only one cluster of *P. falciparum* infections in patients returning from Grande Comore was identified in August 2013 within a Réunion family of Comorian origin. Out of the five people who went on a trip during the winter holiday, four were infected by *P. falciparum* and have their first clinical manifestations in the same 2 days period (the two youngest patients were hospitalized, and the two adults were treated on an outpatient basis). A special investigation was conducted into this disease cluster and revealed the absence of chemoprophylaxis, bed nets, and skin mosquito repellents. Because the family thought the risk was practically zero in the dry season and due to the total cost of anti-malarial for chemoprophylaxis (500 euros), they decided to not buy it, contrary to previous visits.

A microsatellite comparison of the strains in this cluster at the National Malaria Reference Centre was conducted on three out of the four strains available. The parasite load in the fourth patient was too low to yield a result. The analysis showed that two out of the three patients were probably infected by the same strain; the three microsatellites for which results were obtained were completely homologous, the two others microsatellites was not determined due to the poor sensitivity of the method (see Table 3), whereas the third patient was infected by a different strain (four out of five markers).

**Table 3:** Analysis of *P. falciparum* microsatellites of strains taken from the family disease cluster

Case	Parasitaemia	TAA 87	TAA 81	ARA 2	PfPK2	TAA 60	Conclusion
Patient 1	0.002	–	–	–	–	–	
Patient 2	0.067	84	185	105	175	88	Probably homologous strains
Patient 3	0.06	84	–	–	175	88	
Patient 4	0.76	96	179	–	190	82	Different strain

## Discussion

The thoroughness of malaria surveillance in the past 4 years in Réunion was high, and malaria cases were reported by private and public institutions alike by both laboratories and physicians in charge of the patients’ treatment. Though it is possible a case was unreported and treated on an outpatient basis, the surveillance data are representative of the evolution of imported malaria in Réunion during the past few years. Since 2001, the number of imported malaria cases has been consistently dropping in Réunion. The incidence rate reached a new low in 2016. At the same time, the number of travellers from malaria endemic regions has doubled. This decrease could be due to the overall drop in malaria transmission in the islands in the southwestern Indian Ocean; this drop has been quantified both in the Comoros archipelago (Mayotte and the Union of Comoros) and Madagascar [9–11]. Between 1997 and 2010, malaria contracted in Madagascar or Comoros was the number-one cause of disease among travellers in the southwestern Indian Ocean [20]. In Réunion, most malaria cases of imported malaria between 2003 and 2008 were due to travel in Comoros or Madagascar. The incidence rate was highest for travellers returning from Comoros [7]. Currently, the number of imported malaria cases from Comoros has fallen drastically, as has the incidence rate; this change corresponds to a lower transmission rate in every island in the Union of Comoros, especially Anjouan and Moheli, followed by Grande Comore. The significant drop in 2013 and 2014 corresponds to the implementation and completion of the mass drug administration programme in Grande Comoros [9, 21]. Between 2001 and 2011, the largest proportion of severe imported malaria cases in Réunion came from Madagascar according to a study carried out by the island’s intensive care departments [22]. Currently, most imported malaria cases in Réunion come from tourists who have travelled to Madagascar.

Considering the type of trip, excluding medical evacuations, affinity tourism in Madagascar and Comoros was the cause of 65% of imported malaria cases in Réunion between 2013 and 2016. Between 1997 and 2010, malaria was the most common pathology (74% of diagnoses) affecting people in the Indian Ocean region who were travelling to see family or friends according to the surveillance network Geosentinel [20]. Tourism to VFRs remains the main reason for imported malaria cases in Réunion. This is the case in most non-endemic countries [23]. A range of factors could explain this situation, including a lesser degree of vigilance regarding malaria, a lesser use or access to medical advice among travellers, higher-risk travel conditions (rural environments, less enclosed houses, visits to a resident’s home), poorer adherence to protection measures (chemoprophylaxis, use of treated bed nets, individual protection measures), and potentially the cost of preventative measures [24]. Anti-malarial coverage by social security organizations was suggested to decrease malaria rates related to VFRs in England as well as French Guinea, where since 2007 the social security regime has covered one anti-malarial per year for coastal residents visiting family in the country’s interior or along its rivers [25, 26]. The efficacy of these strategies has not yet been clearly shown; other factors doubtless play a role on adherence [24].

The lack of chemoprophylaxis or poor adherence was found in the majority (96%) of malaria cases of imported malaria between 2013 and 2016, regardless of trip type. The various studies conducted in the 2000s found a lack of chemoprophylaxis, poor adherence, or an inappropriate prescription to be common features in imported malaria cases in Réunion. While the lack of chemoprophylaxis is most often due to the absence of medical consultation before travel, there was also an inadequate knowledge of the recommendations, as shown in the study by Di Bernardo et al. Specifically, no chemoprophylaxis was prescribed or an inappropriate prescription was provided in more than 50% of malaria cases [8]. The analysis by the National Malaria Reference Centre of the strains collected in 2013 confirms the prescriptions provided were inappropriate given the anti-malarial resistance in the surrounding region and justifies the recommendations for travellers arriving from Comoros to Madagascar, namely to take an appropriate anti-malarial for Zone 3, i.e. a combination of atovaquone and proguanil, mefloquine, or doxycycline according to the patient’s age and possible medical contra-indications. Chloroquine/proguanil and chloroquine are not indicated [27].

With respect to the treatment of malaria infections, most treatments administered in hospital and non-hospital settings used artemether/lumefantrine or atovaquone/proguanil for uncomplicated infections. In a non-hospital setting, Nivaquine was inappropriately prescribed to treat *P. falciparum* infections in three out of thirty-three patients, or 9% of malaria cases. While we do not have data regarding polymorphisms of molecular markers indicating resistance to artemisinin (*k13*) in the strains collected in Réunion, studies conducted in Grande Comore and Madagascar found that there were no molecular markers indicating resistance to artemisinin derivatives in the surrounding region [28, 29].

With respect to the reemergence of malaria in Réunion, not only has the number of imported malaria cases decreased from year to year, but the number of gametocyte carriers over the past 4 years has remained very low. Most malaria cases occurred outside of areas in which the *Anopheles* mosquito persists or during periods when the density of vector populations has been unfavourable to transmission from an imported case. *Anopheles* mosquitoes were found only seven times with regard to imported malaria cases. Mosquitoes were found around six subjects who were not gametocyte carriers and just once around a subject who was a carrier of *P. falciparum* gametocytes. These data are reassuring with respect to the risk of transmission. Nevertheless, the work of Girod et al. in 1996 and 1997 demonstrated the wide-ranging vectorial capacity of *An. arabiensis* in Réunion. This species has a capacity of eleven in certain areas in the west, meaning the potential to generate eleven malaria cases from a single imported case [3]. Because of the low number of gametocyte carriers and the absence of an *Anopheles* mosquito population when most malaria cases are imported, the risk of the appearance of introduced malaria cases and indigenous malaria cases appears low. It cannot be completely excluded, however, because *Anopheles* mosquitoes were found around a subject who was a gametocyte carrier. Only the elimination of *An. arabiensis* from Réunion would reduce this risk completely. Even then, points of entry to the island would need to be checked to curtail the reintroduction of these mosquitoes from neighbouring islands due to daily air traffic [30–34].

Though it is probably very low, there remains a risk of a temporary reintroduction of malaria to Réunion. With the success of the anti-malaria campaigns in the Comoros archipelago, Madagascar is now the destination that poses the highest risk for imported malaria cases of malaria in Réunion [35–37]. Most malaria cases are related to affinity tourism, as is the case elsewhere in the world. The majority of malaria cases involve travellers who did not take any form of chemoprophylaxis (lack of medical consultation prior to travelling, lack of prescription, non-adherence) and did not follow any personal protection measures to combat vectors. To decrease the number of malaria cases imported into Réunion, prevention measures should specifically target travellers arriving from Madagascar by encouraging tour agencies and airlines operating flights between Madagascar and Réunion to share information about preventing malaria. The goal would be to incite travellers to see their doctor and take chemoprophylaxis. As described above, we found that non-hospital medical practitioners in Réunion lacked proper training with regard to preventing malaria and applying the national recommendations as well as treating the disease. An update could be written by the Indian Ocean Regional Health Agency in collaboration with the Regional Health Professions Union (URPS) and distributed to practitioners throughout the island. Appropriate prescriptions and risk awareness might not be enough to change travellers’ habits due to cultural differences and the cost incurred by families, who are often poor, when purchasing chemoprophylaxis, bed nets, and mosquito repellents. Work with Malagasy associations in Réunion could potentially improve adherence to prevention measures. However, social security authorities should consider covering chemoprophylaxis. If, on a single case basis, the cost of treating a malaria infection is higher than that of providing chemoprophylaxis, the cost-efficiency ratio for all of Réunion should first be assessed.
